# The impact of spatial and social structure on an SIR epidemic on a weighted multilayer network

**DOI:** 10.1007/s10998-021-00440-8

**Published:** 2022-01-06

**Authors:** Ágnes Backhausz, István Z. Kiss, Péter L. Simon

**Affiliations:** 1 Institute of Mathematics, ELTE Eötvös Loránd University, Pázmány Péter sétány 1/c, Budapest, 1117 Hungary; 2 Alfréd Rényi Institute of Matematics, Reáltanoda utca 13-15, Budapest, 1053 Hungary; 3 Department of Mathematics, School of Mathematical and Physical Sciences, University of Sussex, Falmer, Brighton, BN1 9QH United Kingdom; 4 Numerical Analysis and Large Networks Research Group, Hungarian Academy of Sciences, Pázmány Péter sétány 1/c, Budapest, 1117 Hungary

**Keywords:** Multilayer network, SIR epidemics, Household structure, Random graphs, 92D30

## Abstract

A key factor in the transmission of infectious diseases is the structure of disease transmitting contacts. In the context of the current COVID-19 pandemic and with some data based on the Hungarian population we develop a theoretical epidemic model (susceptible-infected-removed, SIR) on a multilayer network. The layers include the Hungarian household structure, with population divided into children, adults and elderly, as well as schools and workplaces, some spatial embedding and community transmission due to sharing communal spaces, service and public spaces. We investigate the sensitivity of the model (via the time evolution and final size of the epidemic) to the different contact layers and we map out the relation between peak prevalence and final epidemic size. When compared to the classic compartmental model and for the same final epidemic size, we find that epidemics on multilayer network lead to higher peak prevalence meaning that the risk of overwhelming the health care system is higher. Based on our model we found that keeping cliques/bubbles in school as isolated as possible has a major effect while closing workplaces had a mild effect as long as workplaces are of relatively small size.

## Introduction

SARS-CoV-2 and the associated disease COVID-19 has had unprecedented worldwide impact placing extraordinary strain on health-care sectors and economies [1] with further negative effect on education and mental health [2]. Typical control measures included full-blown lockdowns in an attempt to suppress the disease [3–9] combined with test and trace programs [10] with various success rates. It has now been clear for some time that elimination of the virus is not possible [11, 12] and with new vaccines on their way, regional or country-wide lockdowns are used to avoid overwhelming health-care systems and to buy more time to learn about the virus, develop better treatments including a new vaccine/vaccines. Since the prevalence in most countries is low, partial or full lockdowns are usually followed by new outbreaks or secondary waves which makes the acceptance of and the compliance with lockdowns more challenging [13].

In most countries there has been a concerted effort to develop country-specific models of disease transmission which are often parameterised with relevant data such as age-structure and mixing between different classes [14, 15], mobility data taken from various sources, existing or new surveys/apps, school [16] and workplace sizes and locations etc. Models range from simplistic mass action models, with some extended to include age-structure and realistic incubation and infectious times, and multi-layer network models [17] with realistic demographic data to country-wide individual-based simulation models that ingest a large amount of static and dynamic population data [18].

Typically, in most countries scientific evidence for policy making is taken from a range of models since the uncertainty in most models is high. Any model be it simple or complicated can be challenged. Simple models tend to lack realism and neglect many features of the population (e.g. age structure, mixing) and disease (e.g. non-exponential incubation and infectious periods [19]), but they rely on fewer parameters and are more transparent. Complex models need huge amount of data for parameterisation, which is not always available and assumptions need to be made. While they tend to be more realistic, such models are expensive to run and make scenario testing and parameter inference difficult and are less transparent, often changes in output are difficult to correlate with changes in input parameters.

In this paper we will use a multilayer network [20] to capture features of contact patterns which are known to be relevant for the transmission of COVID-19 and other infectious disease. These are: (1) household structure, (2) schools for children and workplaces for adults, (3) spatial structure/embedding, and (4) casual contacts (e.g. contacts realised during shopping, going to the GP, communal places/playgrounds etc). We adapt some of these components to Hungary, in particular the statistics of the household structure. We then run a susceptible-infected-recovered (SIR) model on this network and study the impact of interventions such as closing schools, work or public places. The infection rates in different layers and the sizes of schools and work places are varied systematically and their effect on the system behaviour is studied. First, we investigate the time dependence of the number of infected and recovered individuals as the values of the different parameters are changed. Then we focus on the final epidemic size and the peak of the epidemic and study how the parameters affect their values. It is important to understand how a given final epidemic size can be achieved with the smallest possible peak and relate this to the structural properties of the network.

The paper is structured as follows. In Sect. 2 we describe the construction of the underlying network, the epidemic dynamics and enumerate parameters whose values can be found /fixed and those that are subject to investigation. Section 3 contains a systematic investigation of the impact of parameter values on the time dependence of the number of infected and recovered nodes, while in Sect. 4 we study the relation of the peak epidemic prevalence and the final epidemic size. We also provide some analytical insight into the relation between the values of peak epidemic prevalence and final epidemic size for the standard SIR model and explore how this relation changes in the network setting. Finally, in Sect. 5 we discuss the implications of our findings.

## Model

In this paper the population and interaction between members of the population is described by a network where each individual is represented by a node and disease-transmitting contacts are encoded by links/edges between nodes. We mimick some of the relevant features of realistic contact patterns such as households, schools, workplaces and other casual contacts, such as shopping or visiting the doctor. These will be implemented as multiple layers over the same nodes, leading to a network with four layers.

The population is divided into three age groups, children/young people, adults and elderly people. They will be grouped into households according to the Hungarian data as it will be specified later. The members of the population spend different times in different layers of the network and the strengths of the contacts are also different, hence the transition rates in different layers are different. This will be taken into account by allocating different weights on the links in the layers. These weights will be parameters of our model.

### The four layers of the network

Our network consists of four layers: (1) households, (2) schools and workplaces, (3) spatial structure, and (4) community places (e.g. shopping or medical centers, etc.)

**Households** are the basic units of our network. It is taken into account that the sizes of the households and the ages of its members are different. Based on the data available from the Hungarian Statistical Office (http://www.ksh.hu/nepszamlalas/docs/tablak/lakas/06_01_02_14.xls), we created a list of 16 household types, these are the most frequently occurring in Hungary. For example, households with two adults and two children or households containing two elderly people. The frequency of each type was also determined from the data. The number of households is a parameter of our model. Once this number is given, we choose the different types from this distribution. Each household is considered to be a complete graph with a given edge weight, denoted by $\tau _\mathrm{h}$, which corresponds to the per-contact relative infection rate within households.

The layer of **workplaces and schools** contains links between adults and children. All workplaces are complete graphs with the same size and with edge weights $\tau _\mathrm{wp}$. The adult population is divided into groups of the same size randomly, independently of the other layers, such that each configuration has the same probability. Similarly, children population is divided into schools randomly, independently of the other layers. All schools have the same fixed size. Schools themselves have two layers: small complete graphs of larger weight, called cliques, representing the group of children who spend much of their time together, and an underlying complete graph connecting every pair of students with a smaller weight. This represents the chance of infection at lunch or sport activities, where any two students can meet each other. The size of cliques will usually be 10. This is in accordance with the observation that children spend most of their time in school in a well-defined small group of size around 6–10. (We note that detailed data is collected from volunteers by a Hungarian institution about the connections of people in different age groups, this data is available at https://covid.sed.hu/tabs/response.) We will have two parameters here. First, $\tau _\mathrm{cls}$ is the proportion of the infection parameter within a clique and within a household. This will usually be chosen to 1, as sitting close to each other in the classroom can have the same effect as living in the same household. On the other hand, $\tau _\mathrm{sch}$ will be the proportion of the infection parameter between and within school cliques. For example, $\tau _\mathrm{sch}=20\%$ means that the probability that a children infects someone from the school outside his clique is $20\%$ of the probability that he infects someone from the same clique. The case $\tau _\mathrm{sch}=0$ means that the school consists of perfectly separated cliques of fixed size. The formulation of cliques within a school is uniform random, every configuration has the same probability, and this is independent of any other schools or layers.

The **spatial or geometric structure** is represented by an arbitrary graph, the nodes of which are the households. If two households are connected with a link then every member of both household is connected to every member of the other one. The weights of these edges are denoted by $\tau _\mathrm{g}$ representing the relative infection rate between neighbours. In all our simulations, we choose this graph to be a lattice describing a province-like residental area, that can be tuned to be more town-like by adding diagonal edges to the lattice in the middle part of the domain. This represents block of flats and hence makes the network denser.

The fourth layer of the network is given by **community/public places** such as shops, playgrounds, medical centres etc. These belong to different residential areas. The community places are also work places (e.g. for doctors and nurses), so these are chosen randomly from workplaces. Their number is calculated as follows. The parameter of the model is the size of this places, i.e. the number of people visiting each of them. For example, if there are $N=10000$ people in the population and the size of community places is chosen to be 200, then their number is $10000/200=50$. Hence, 50 of the already existing workplaces is chosen randomly and their members are considered to be the employees in these community places. In the next step, the whole population is divided into 50 clusters by using the K-means clustering algorithm. The members of these clusters are those who visit the same community place, e.g. a shopping centre. Each member of this cluster is linked to each employee of the corresponding workplace, forming a star-like graph. The members are not connected to each other, they can infect each other only through those people who work there. The edge weights on these links are denoted by $\tau _\mathrm{s}$. We note that one resident in a cluster is connected to only one community place.

**Fig. 1:**
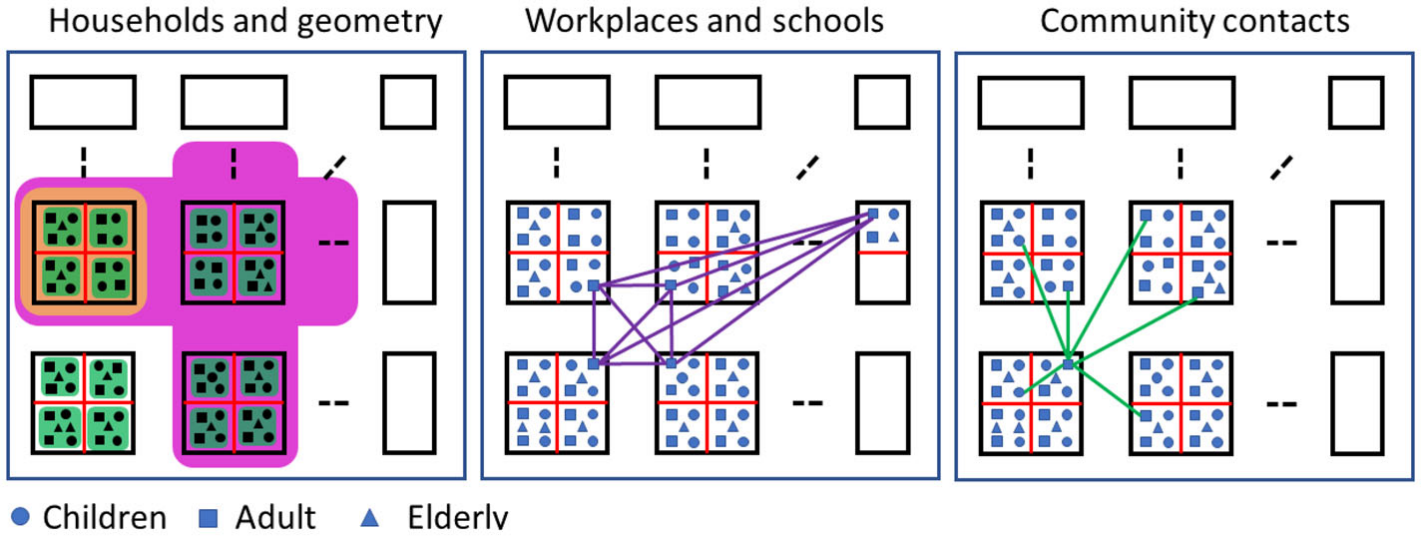
A caricature of the multilayer network where the building blocks are households (see green sets in the left panel) which are placed on or act as nodes in a square lattice which mimics spatial proximity. Households in neighbouring lattice nodes can be still connected, for example modelling a block of flats (see orange set in the left panel). Connections between these larger units to close neigbours are still possible (see pink set in the left panel). Workplaces and schools are formed by creating groups of adults and children, respectively. These are created independently of the position of their households on the network (see middle panel with a workplace example, schools are formed in a similar way). Finally, community contacts are created whereby individuals from a small percentage of workplaces are selected to act as star nodes where the spokes are found by a K-means clustering algorithm based on proximity or distance from the star nodes, see the right panel. (Color figure online)

Finally, we get an adjacency matrix in each layer, where we can have different weights on the edges. These weights are stored in the corresponding adjacency matrices. The adjacency matrix of the whole multilayer network is the sum of these four matrices. The structure of the layers is shown in Fig. 1.

### The parameters of the multilayer network

Here we summarise all parameters of our model.

- The total number of households together with their type and frequency.
- The adjacency matrix of households, representing the spatial structure. In the present paper this is a lattice with a possibly denser part in the middle. Its parameters are the horizontal and vertical sizes of the lattice and proportion of households belonging to the denser part.
- The sizes of the workplaces and the proportion of workplaces that are closed.
- The sizes of cliques in schools and the number of cliques in a school. (Their product yields the school size.)
- The capacity of a community place, i.e. the number of people visiting a place.
- The edge weights, i.e. the relative infection rates in the layers:
  - The weights of edges within households, $\tau _\mathrm{h}$,
  - The weights of edges between households, $\tau _\mathrm{g}$,
  - The weights of edges within workplaces, $\tau _\mathrm{wp}$,
  - The weights of edges within school cliques, $\tau _\mathrm{cls}$ (if this is zero, then schools are closed),
  - The ratio of weights of school edges to within clique edges, $\tau _\mathrm{sch}$,
  - The weights of edges in the layer of community places, $\tau _\mathrm{st}$.

The numerical experiments below use the following values of the parameters as default values. It will be indicated where one or more of these values are modified.

The network of households is a $40\times 40$ size lattice and the proportion of households lying in the denser region is $20\%$. There are 16 types of households in our model. Their configurations and their proportion among all households is given in Table 1. For each configuration, the triples give the numbers of children, adults and elderly people, respectively.

**Table 1:** Household composition and frequency in Hungary, based on data from http://www.ksh.hu/nepszamlalas/docs/tablak/lakas/06_01_02_14.xls. We note that the average household size is 2.43

Configuration	0, 1, 0	0, 2, 0	0, 3, 0	0, 0, 1	0, 0, 2	1, 1, 0	1, 2, 0	2, 2, 0
Frequency	0,17	0,12	0,04	0,12	0,07	0,04	0,09	0,12
Configuration	3, 2, 0	4, 2, 0	1, 0, 1	0, 1, 1	0, 2, 1	1, 1, 1	1, 2, 1	2, 2, 1
Frequency	0,05	0,02	0,01	0,07	0,04	0,01	0,02	0,01

Furthermore, we have:

- The size of the workplaces is 10 and the proportion of closed workplaces is 0,
- The size of cliques in schools is 10 and the number of cliques in a school is 20,
- The capacity of a community place is 200.

We assume that the largest relative infection rate is the one within households. This will be taken to be 1 and the other infection rates are between 0 and 1. We note that an overall infection rate is applied in the whole network representing the strength of the infection. Its default value is $\tau =0.1$. This value multiplies all edge weights in each layer. The default values of the relative infection rates are: $\tau _\mathrm{h}=1$, $\tau _\mathrm{wp}=1$, $\tau _\mathrm{cls}=1$, $\tau _\mathrm{sch}=1/5$, $\tau _\mathrm{g}=1/10$ and $\tau _\mathrm{s}=1/10$.

### The epidemic model

We apply the standard SIR model for describing the propagation of the infection. Each member of the population is represented by a node of the network and each node can be in one of three states: susceptible (S), infected (and infectious) (I) and recovered (R).

The change of state for each node is described by independent Poisson processes the rate of which depends on the states of neighbouring nodes. The rate of infection of a given node, i.e. transition from S to I, is the sum of the weights on edges (also called relative infection rates) ending at that node multiplied by the overall infection rate $\tau$ representing the strength of the infection. The weight of the edge depends on the layer where the edge is lying. The rate of recovery, i.e. transition from I to R, is $\gamma$ for each node. (For simplicity, we assume that this is identical at each node.) As it is well known, $1/\gamma$ is the average time spent in state I.

Since each node can be in three different states, the whole network can be in one of $3^N$ states, where *N* is the number of vertices in the network. The evolution of the whole network is described by a Markov chain on a state space of dimension $3^N$. The time dependence of the probabilities of the states is governed by the master equation that is a system of $3^N$ linear ordinary differential equations. Since this system cannot be handled because of its size, we run Gillespie simulations on the network. We note that population level mean-field approximations can also be used for random networks. However, our multilayer networks have a non-random structure, hence the differential equation approximations cannot capture the detailed behaviour of the system. In this paper, we do not investigate the accuracy of these approximations [21].

An individual-based stochastic Gillespie simulation is run as follows. We start the network from a random state with a given number of infected nodes, the remaining nodes are taken to be susceptible. At each iteration or update the rate of transition of each node is determined based on the given state of the network. The time to the next event then is chosen from an exponential distribution with the parameter being the sum of all the transition rates. Then according to the Gillespie algorithm a node is chosen at random but proportionally to the value of its transition rate. This step is repeated until there are no infected nodes in the system. At the end of the process, all nodes are either recovered or susceptible. The number of recovered is called the final epidemic size. The result of a run is a series of time points, where the state of a node changed and the values of S, I and R at those time points. Thus we can plot the time dependence of S, I and R for each run, or we can plot the average of those for several simulations.

## Results: the impact of contact layers on the time course of the epidemic

In this section we perform computer simulations to study the effect of the different parameters of our model, such as the relative infection rates in different contact layers and the sizes of schools and workplaces. Since some of these parameters can be controlled (e.g. with closing some of the schools or workplaces, or decreasing the frequency of going to stores by a partial curfew), this may lead to useful conclusions about the effectiveness of different social distancing strategies.

The stochastic simulations are run on several networks in this section. For each parameter set, we randomised 4 graphs with the given parameters and run 6 Gillespie simulations on each of them. Then the average of these 24 runs is shown in the Figures.

### Household distribution

**Fig. 2:**
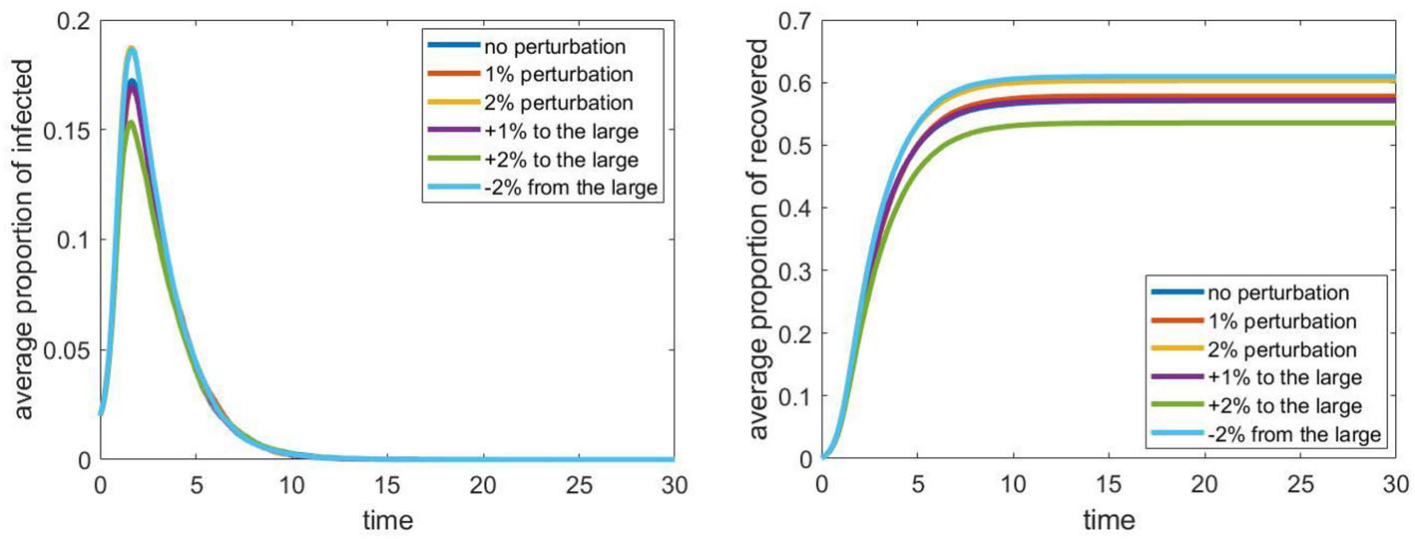
The evolution of the proportion of infected and recovered with the different perturbations of the household distribution (average of 24 runs). The parameters are fixed according to Sect. 2.2, the household network is a grid of size $40\times 40$, the population size *N* is approximately 4000

Since we used only estimated data about the distribution of the number and ages of people living together in a household, we examine the effect of small changes in this input data. We simply added perturbations of 1–2% to the original frequency vector (either at random, or by increasing/decreasing the weight of the more frequent configurations), see Table 1. In Fig. 2 we can see the average of 24 simulations as explained above. We can see that the difference in the outcome has approximately the same magnitude as the perturbation. However, the main characteristics of the process remain the same. Hence, the error coming from rounding the proportions of different configurations most likely does not lead to absolute errors of more than 1–2%.

### Effect of infections at school

**Fig. 3:**
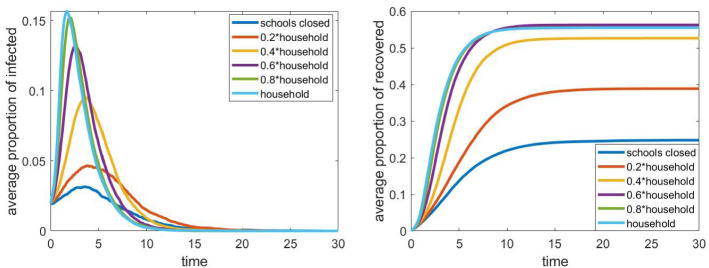
The evolution of the proportion of infected and recovered with different school clique relative infection rates $\tau _\mathrm{cls}$. Other parameters are fixed according to Sect. 2.2, the household network is a grid of size $40\times 40$, the population size *N* is approximately 4000

Next we examine how the intensity of infections at school affects the spread of the epidemic. First, we changed the parameter $\tau _\mathrm{cls}$, which describes the intensity of infections within the cliques of size 10, and which also has an effect on the intensity of infections between cliques, as the latter is $20\%$ of $\tau _\mathrm{cls}$ in the baseline scenario. The case $\tau _\mathrm{cls}=0$ corresponds to the case of closed schools, while $\tau _\mathrm{cls}=1$ means that the probability of infection within a small clique at school is the same as in the households. Again, the number of households was 1600, arranged in a $40\times 40$ grid, which means a population of approximately 4000 individuals. For each parameter $\tau _\mathrm{cls}$, we run 24 simulations as explained above. The results presented in Fig. 3 are obtained as the average of the 24 different curves.

We can see that this parameter has a significant effect on the epidemic: With all schools open, and $\tau _\mathrm{cls}=1$, the maximal proportion of infected is approximately five times the corresponding value with closed schools. This means that closing schools seems to be an effective social-distancing strategy in our model. Between the two extremes, i.e. closing all schools or leave all of them open, the value of the peak epidemic and the total proportion of recovered individuals are both monotone functions of the intensity, as expected.

**Fig. 4:**
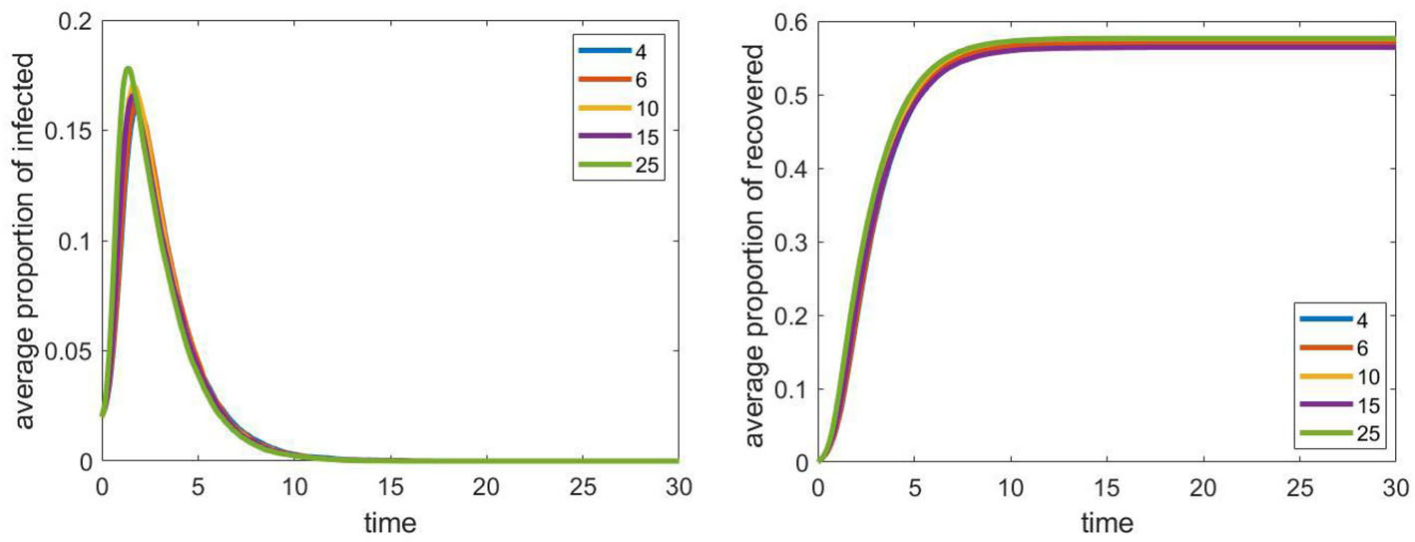
The evolution of the proportion of infected and recovered individuals for different clique sizes with fixed total size of the school (200 students). Other parameters are fixed according to Sect. 2.2, the household network is a grid of size $40\times 40$, the population size *N* is approximately 4000

Our next question is about the effect of the internal structure of schools on the spread of the epidemic. Now we increased the size of cliques within the schools. At the same time, we decreased the number of cliques in each school, so that their total size (200 students in each of the five schools) remains fixed. Results can be seen in Fig. 4. All other parameters and the number of iterations were same as in the previous case.

We can see that under these conditions, the sizes of the cliques do not have a significant impact on the evolution of the process. We remark that when we run the simulations on a smaller graph, the effect of the clique size was more noticeable, and we could also see the monotonicity property: larger clique size meant larger epidemics.

**Fig. 5:**
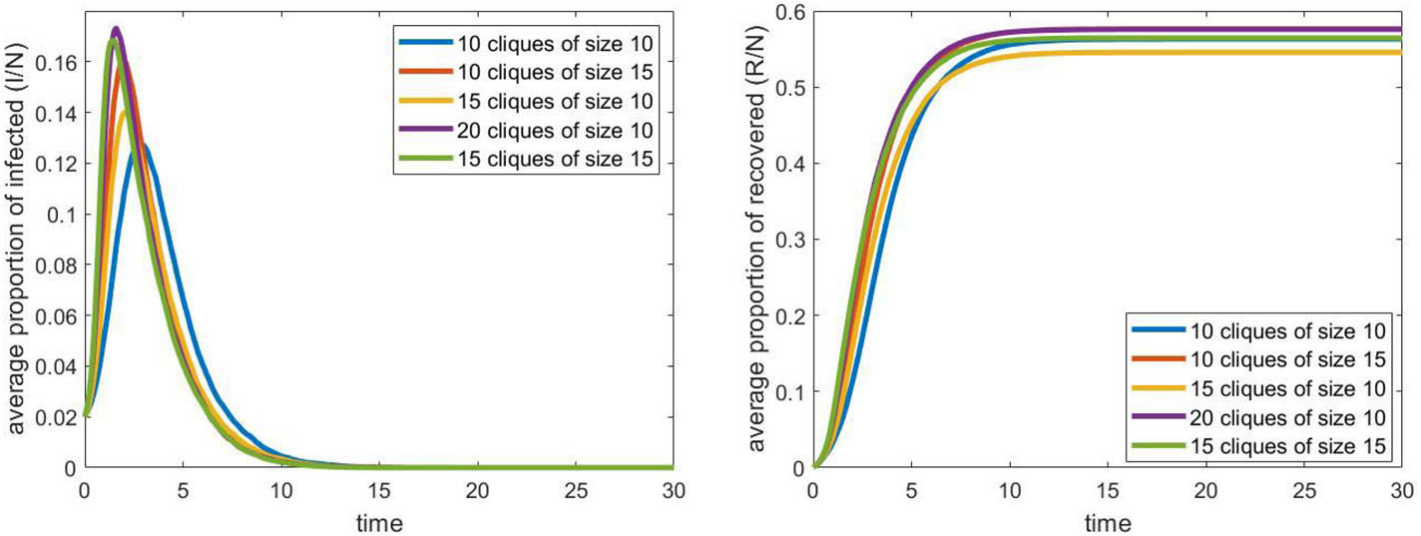
The evolution of the proportion of infected and recovered individuals for different values of the total size of the school and different clique sizes

Going further in this direction, we compared different school sizes with different clique sizes. The results can be seen in Fig. 5. In this setup, we see that the total size of the schools can have a significant effect on the maximum number of infected individuals: schools with 200 students lead to an increase of approximately $40\%$. Here we see that clique sizes can have larger effect in the case when schools are smaller (150 students instead of the original 200).

**Fig. 6:**
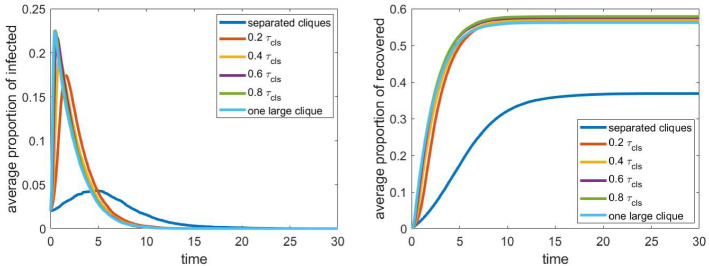
The evolution of the proportion of infected and recovered individuals for different values of the relative infection rate between school cliques ($\tau _\mathrm{sch}$)

Finally, we examine the effect of the relative infection rate between the cliques in the schools, that is, the sensitivity of the model to the parameter $\tau _\mathrm{sch}$. In this case, one of the extreme cases is $\tau _\mathrm{sch}=0$, when the cliques are completely separated from each other. The other extreme is $\tau _\mathrm{sch}=1$, when the relative infection rate is the same within and between groups, that is, the school consists of one large clique. As we can see in Fig. 6, this parameter has a significant effect with a five fold difference in the value of peak epidemic. In addition, the difference between $\tau _\mathrm{sch}=0$ and $\tau _\mathrm{sch}=0.2$ is much larger than the difference between the latter and the case of $\tau _\mathrm{sch}=1$, when connection between any two students is as strong as household connections. Therefore, even a weak connection of students belonging to different classes can lead to a strong epidemic wave, and keeping different school classes isolated, or in a bubble, seems to be an effective control measure.

### The effect of infection at work

**Fig. 7:**
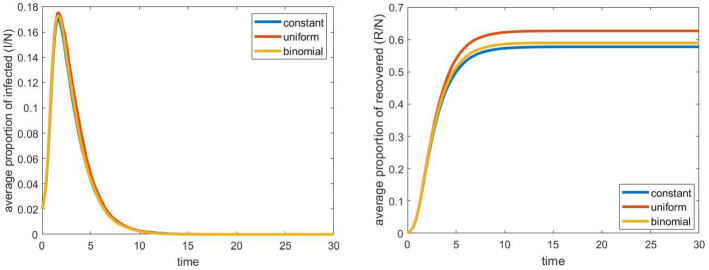
The evolution of infected and recovered with different workplace size distributions: constant 10, uniform from $\{1, 2, \ldots , 19\}$, and binomial with $n=40$ and $p=0.25$. Other parameters are fixed according to Sect. 2.2, the household network is a grid of size $40\times 40$, the population size *N* is approximately 4000

In this section we examine the effect of infections at workplaces. All other parameters (including the number of iterations of randomizing the graph and the Gillespie algorithm) are the same as in the previous section.

Before studying the effect of the different parameters, it is worth examining the role of equal workplace sizes. More precisely, in this first simulation, we compare the evolution of the epidemic when the workplace size is a fixed constant, and when it is chosen randomly. We have chosen two probability distributions with mean 10, which is the fixed size in the constant case. In particular, we run simulations in the case when the workplace size is chosen uniformly at random from the set $\{1, 2, \ldots , 19\}$, and when it has binomial distribution with parameters $n=40$ and $p=0.25$ (this can be viewed as a distribution “between” the deterministic and the uniform one). As Fig. 7 shows, until the distribution is bounded and does not have a large variance, the results are very similar to the deterministic case. In the sequel, we continue with the latter, where all workplace sizes are fixed and equal to each other.

**Fig. 8:**
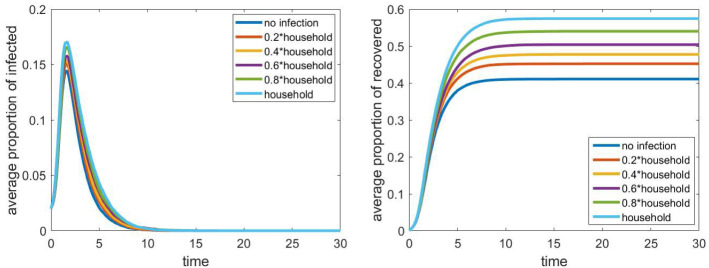
The evolution of infected and recovered with different workplace relative infection rates $\tau _\mathrm{wp}$. Other parameters are fixed according to Sect. 2.2, the household network is a grid of size $40\times 40$, the population size *N* is approximately 4000

Figure 8 shows the effect of the relative infection rate $\tau _\mathrm{wp}$. The model is not too sensitive with respect to the values of this parameter, however, we can see some differences and monotonicity in the proportion of recovered individuals. Notice that the workplace infection intensity could be as large as the infection intensity at home, and this did not lead to a significant difference in the epidemic spread. This emphasises the importance of stabilising the groups of people who meet each other regularly: in our model, workplaces are of size ten, but their members were always the same. This is similar to the case where the school cliques are completely separated, and we could see that this can be an effective control strategy with group of size 10.

**Fig. 9:**
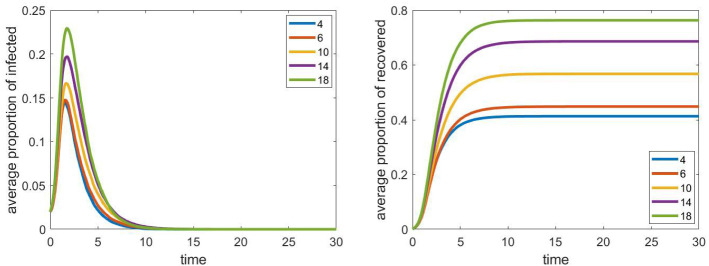
Evolution of the proportion of infected and recovered with different workplace sizes. Other parameters are fixed according to Sect. 2.2, the household network is a grid of size $40\times 40$, the population size *N* is approximately 4000

In Fig. 9, we can see the dependence of the spread of the epidemic on the size of the workplaces. The model is moderately sensitive to this parameter; groups of size 18 instead of size 4 lead an increase of a factor of two in the total proportion of recovered people. Compared to the case of schools, the effect of this parameter is much more significant. This may be due to the fact that public places are also workplaces in our model, and if the infection can spread within a larger group of employees, then they can cause a huge number of infection among the customers. Hence, as we already assumed a weak connection between any two student of a school, strengthening some of these by increasing the number of cliques may have a smaller effect than increasing the probability that the infection spreads at a public place with more people.

**Fig. 10:**
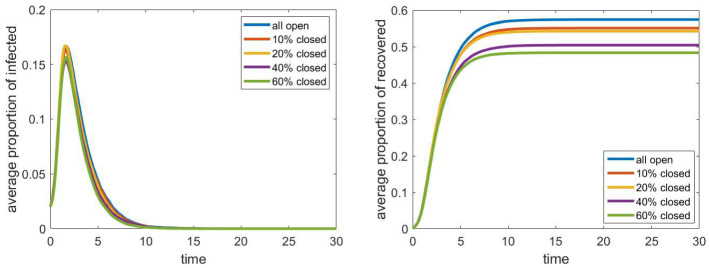
Evolution of the proportion of infected and recovered after partially closing workplace. Other parameters are fixed according to Sect. 2.2, the household network is a grid of size $40\times 40$, the population size *N* is approximately 4000

Finally, in Fig. 10 we see how the partial closure of workplaces affects the spread of the epidemic. It turns out that the differences are not significant. This seems to imply that workplaces which are completely separated from other groups (other than households) do not have an important role in the process. However, we assumed that the public places (stores, medical centres etc.) cannot be closed, and in our model, this represents $40\%$ of the workplaces. As the employees of these have much more contact with other people, these can have a more significant effect. On the other hand, the closure of all workplaces is equivalent to setting the infection parameter $\tau _\mathrm{wp}$ to 0, which had some effect, as we could see in Fig. 8.

To sum up, we note that the size of workplaces has the most significant impact followed by the relative infection rate (edge weight) in workplaces. Closing workplaces that are separated from other people does not seem to be an effective control measure.

### Effect of public or community places

As for public places, we considered two parameters: the capacity (number of visitors) of the units (e.g. stores), and the intensity of infection, measured by the relative infection rate $\tau _\mathrm{s}$; if $\tau _\mathrm{s}=1$, then the rate of infection in public paces and households is the same.

**Fig. 11:**
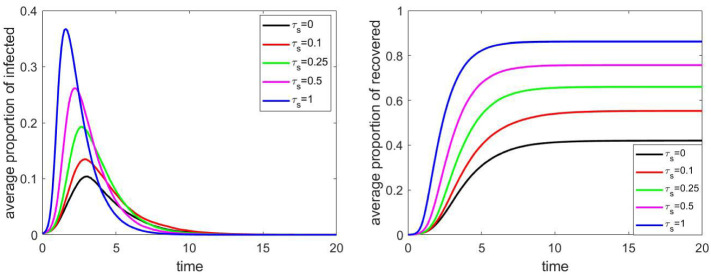
The proportion of infected and recovered individuals for different relative infection rates $\tau _\mathrm{s}$ corresponding to public services. Other parameters are fixed according to Sect. 2.2, the household network is a grid of size $40\times 40$, the population size *N* is approximately 4000

Figure 11 illustrates the effect of $\tau _\mathrm{s}$ on a network consisting of approximately 4000 vertices. We can see that this parameter can have a significant effect only if the intensity of infection in public places and within the households are approximately equal to each other, which does not seem to be the case in real life. Assuming that the infection intensity is $25\%$ or $20\%$ of the intensity within households, then this parameter does not have a notable effect on the evolution of the epidemic spread.

### The effect of geometric structure

Concerning the geometric structure, we had two parameters: the adjacency matrix of the households, and the relative infection rate between neighbouring households, $\tau _\mathrm{g}\in [0,1]$. The value $\tau _\mathrm{g}=1$ means that members of households next to each other have the same probability to infect each other as members of the same household.

**Fig. 12:**
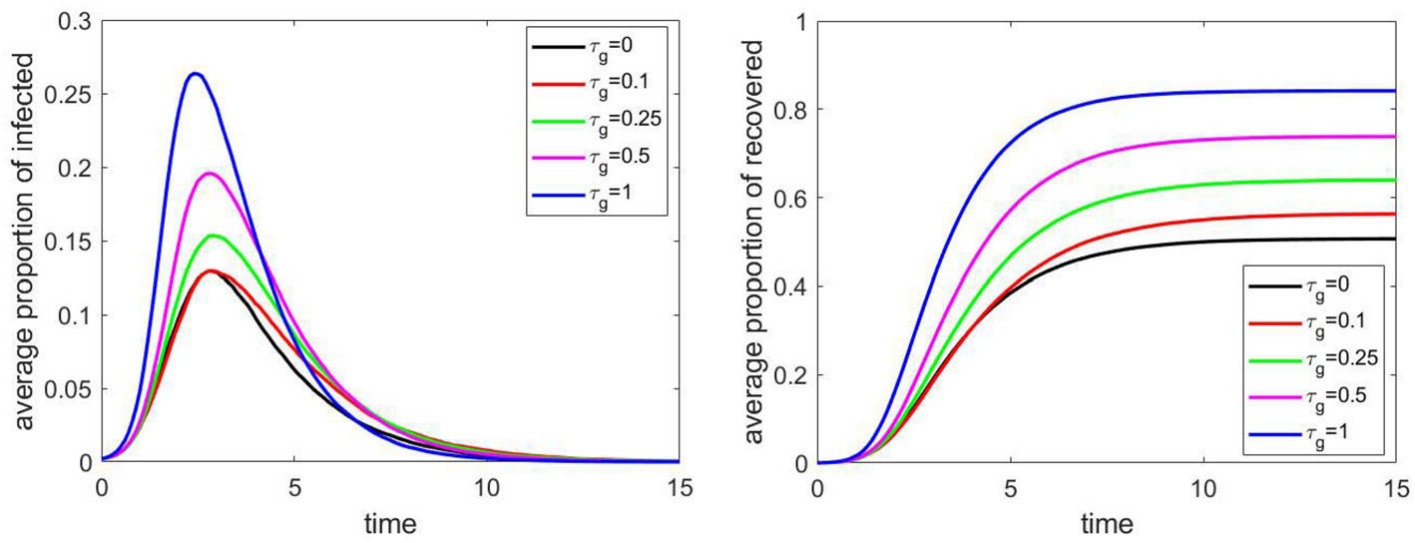
The proportion of infected and recovered individuals for different values of relative infection rate $\tau _\mathrm{g}$ between neighbouring households. Other parameters are fixed according to Sect. 2.2, the household network is a grid of size $40\times 40$, the population size *N* is approximately 4000

The effect of parameter $\tau _{g}$ can be seen in Fig. 12, on a network of size 4000. We have similar conclusions as in the previous case, for public places: we see monotonicity, and the geometric structure has a significant effect only if the relative infection rate is at least $25\%$ that of within households.

### The effect of the size of the population

**Fig. 13:**
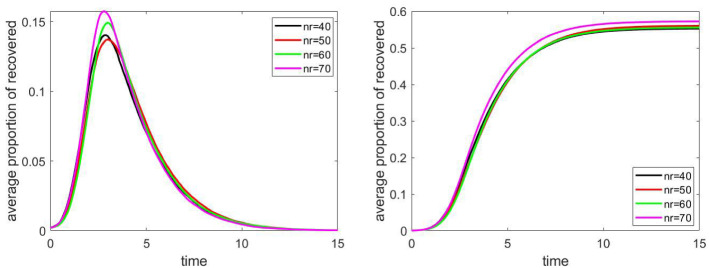
The evolution of the proportion of infected and recovered individuals by changing the size of the population ($n_r$ is the size of the grid); the size of the population is approximately 4000, 6000, 8500 and 12000 in the four cases respectively

By increasing the size of the population, the running time of the computer simulation also increases. Hence we compare the epidemic process on networks of the same structure, but different size. In particular, the model described in Sect. 2.1 was studied, from $40\times 40$ to $70\times 70$ grids. This is the graph of households, with the dense middle part. The number of individuals was approximately 4000, 6000, 8500 and 12000. The effect of this parameter can be seen in Fig. 13. Since the effect is not significant, the conclusions formulated above based on the simulations on $40\times 40$ grids presumably hold for other reasonable population sizes as well.

## Results: peak and final epidemic size

We have seen that the model parameters have significant effect on the time dependence of the number of susceptible and infected nodes. However, in order to understand the effect of the different parameters, it is useful to investigate important variables that have decisive role in the model. The widely used and important variables that we will consider here, are

- $R(\infty )$, the final epidemic size yielding the number of recovered nodes at the end of the epidemic, and
- $\max _t {I(t)}$, the maximal number of infected nodes during the process.

We note that there are other important quantities, for example, the number of daily new cases, that will not be considered here. The significance of $R(\infty )$ originates from the fact that the probability of a possible second wave of the epidemic depends strongly on the number of people affected by the first wave [13, 14]. The maximal number of infected individuals during the process determines the load on the health care system. Hence, when controlling an epidemic is not straightforward and where many individuals will be affected by the end of the epidemic, it is beneficial to at least control the value of the peak prevalence so that the health care sector is not overwhelmed. Therefore, we are interested to map out and understand how the relation between peak prevalence and final epidemic size is affected by an individual or combination of layers in our multilayer network. For example, given a final epidemic size we are interested in what is the range of achievable peak prevalence values.

First, we revisit the relation of the final epidemic size and the maximal number of infected nodes in the simple compartmental model. Then we investigate numerically how these quantities are related in the network model as the different parameters are varied.

### The relation of the final size and peak prevalence in the compartmental model

Let us consider the SIR compartmental model in its usual form.

4.1 $$\begin{aligned} \dot{S}(t)&= - \beta I(t)\frac{S(t)}{N}, \end{aligned}$$

4.2 $$\begin{aligned} \dot{I}(t)&= \beta I(t)\frac{S(t)}{N}- \gamma I(t) , \end{aligned}$$

4.3 $$\begin{aligned} \dot{R}(t)&= \gamma I(t) , \end{aligned}$$

where $\beta$ and $\gamma$ denote the infection and recovery rates and *N* is the size of the population.

It is known that this model yields a relation between the final epidemic size and the maximal number of infected nodes, however, for the sake of completeness we present here a simple derivation of this formula.

Observe that writing the first equation in the form $\dot{S}(t) + \frac{\beta }{N} S(t)I(t)=0$ and multiplying with the integrating factor $\exp \bigl (\frac{\beta }{N\gamma }R(t)\bigr )$ yields that the quantity $S(t)\exp \bigl (\frac{\beta }{N\gamma }R(t)\bigr )$ is constant in time. The value of this constant is given by the initial condition. Assuming $R(0)=0$ and using that $S(\infty )+R(\infty )=N$ we obtain the implicit equation

4.4 $$\begin{aligned} N-R_{\infty } = S(0) \exp \left( -\frac{\beta }{N\gamma } R_{\infty }\right) , \end{aligned}$$

for $R(\infty )$. Applying the approximation $S(0)=N$ we get that the ratio $x=R_{\infty }/N$ satisfies the equation

4.5 $$\begin{aligned} 1-x=\text{ e}^{-xR_0} , \end{aligned}$$

where $R_0=\beta /\gamma$ is the basic reproductive ratio.

Let us turn to deriving the maximal number of infected nodes, $I_{max }=\max _t I(t)$. Equation (4.2) implies that $S=N/R_0$ holds when *I* is maximal. On the other hand, using the first integral

$$\begin{aligned} S(t)\exp \left( \frac{\beta }{N\gamma }R(t)\right) = S(0)=N, \end{aligned}$$

we have $R_0R/N=\ln (N/S)=\ln (R_0)$ at the maximum of *I*. Hence

$$\begin{aligned} I_{max }=N-S-R=N-\frac{N}{R_0} - \frac{N}{R_0} \ln (R_0). \end{aligned}$$

Thus the ratio $y=I_{max }/N$ satisfies the equation

4.6 $$\begin{aligned} y=1-\frac{1+\ln (R_0)}{R_0} . \end{aligned}$$

**Fig. 14:**
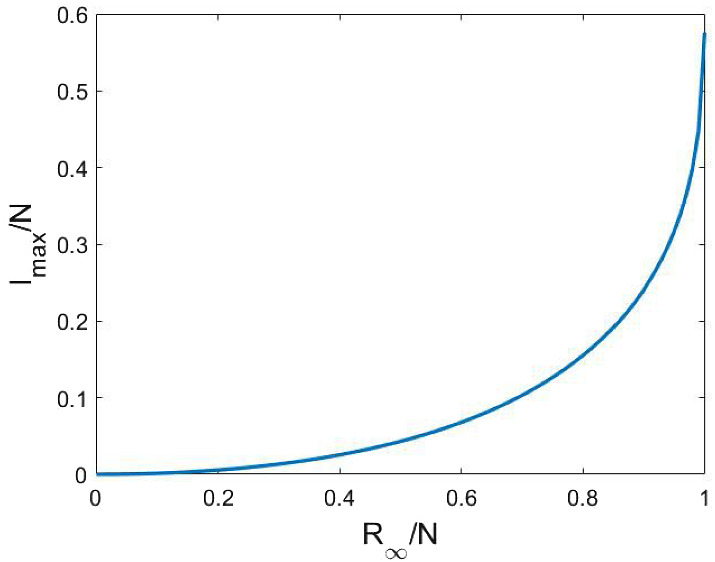
The relation of $R_{\infty }/N$ and $I_{max }/N$ in the compartmental model, (4.1)-(4.3). (Color figure online)

This way, we have derived a relation between *x* and *y*, that is between $R(\infty )$ and $\max _{t} I(t)$ via $R_0$. Namely, we can solve (4.5) for $R_0$ as

$$\begin{aligned} R_0=-\frac{\ln (1-x)}{x} \end{aligned}$$

and then substitute this expression for $R_0$ in (4.6). The relation is shown in Fig. 14. We can see that e.g. for a final epidemic size of $80\%$ the peak prevalence is around $15\%$. It can be verified easily that this relation is monotone, that is an increase in the value of $R(\infty )$ leads to an increasing value of $I_{max }$.

### The relation of the final size and peak prevalence in the network model

Now we run individual based stochastic simulations on the network given in Sect. 2 and determine the values of $R(\infty )$ and $I_{max }$ for different values of the model parameters. We will plot the obtained pairs $(R(\infty ), I_{max })$ together with the curve in Fig. 14 and compare the pairs obtained from the stochastic simulation to those obtained from the compartmental model.

For a given parameter set we generate 10 networks and run stochastic simulations on these until the epidemic ended. Then we determine the final epidemic size $R(\infty )$ and the maximum value of the number of infected nodes $I_{max }$. Each pair $(R(\infty ), I_{max })$ will be plotted with the same colour in the Figures. The average of the 10 pairs will also be shown (with a black diamond).

#### Effect of infection at schools

**Fig. 15:**
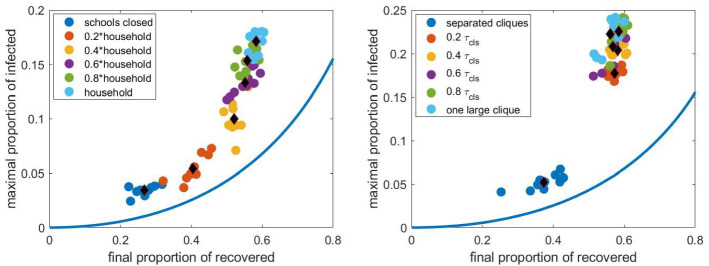
The pairs $(R(\infty ), I_{max })$ obtained from stochastic simulation as $\tau _\mathrm{cls}$ (left panel) and $\tau _\mathrm{sch}$ (right panel) is varied. The average of the 10 pairs computed for a given network is shown with a black diamond. Other parameters are fixed according to Sect. 2.2, the household network is a grid of size $40\times 40$, the population size *N* is approximately 4000. The curve obtained from the compartmental model and shown in Fig. 14 is shown with continuous blue line. (Color figure online)

First, we investigate the effect of the parameters $\tau _\mathrm{cls}$ and $\tau _\mathrm{sch}$ representing the relative infection rate within school cliques and its ratio to the relative infection rate within schools. The left panel in Fig. 15 shows the $(R(\infty ), I_{max })$ pairs for 10 networks as $\tau _\mathrm{cls}$ is varied from zero, corresponding to closed schools, to $\tau _\mathrm{_h}$, the relative infection rate at homes. We can observe that the averages (black diamonds) form a curve similar to that given by the compartmental model, however, lying to left of this and with higher values of $I_{max }$. The maximum value of *I*, belonging to a given final size, is roughly three times larger in the network compared to the compartmental model.

The right panel in Fig. 15 shows these pairs as $\tau _\mathrm{sch}$ is varied from zero, corresponding to schools containing separated cliques, to $\tau _\mathrm{cls}$, corresponding to a school given by a fully connected graph. The curve formed by the averages (the black diamonds) is not as obvious as in the previous case and we can observe that the setup corresponding to separated cliques is quite far from the other scenarios. Increasing the within school infection rate above 20 $\%$ does not increase the final size, but increases $I_{max }$ significantly. Thus we can say that separating the cliques within a school prevents the spread of the infection more effectively than decreasing the relative infection rate within cliques.

Since we have seen in Fig. 4 that the clique size does not have a strong effect on the propagation, we do not consider this parameter here.

#### Effect of infection at workplaces

**Fig. 16:**
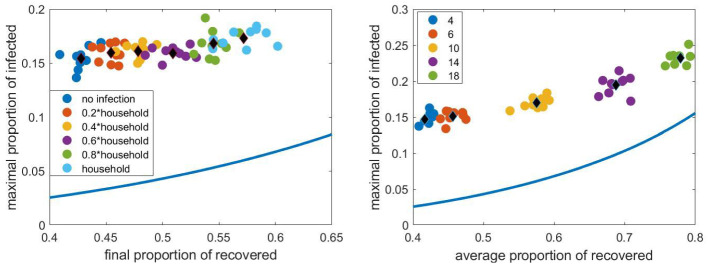
The pairs $(R(\infty ), \max I)$ obtained from stochastic simulation as $\tau _\mathrm{cwp}$ (left panel) and the size of work places (right panel) is varied. The average of the 10 pairs computed for a given network is shown with a black diamond. Other parameters are fixed according to Sect. 2.2, the household network is a grid of size $40\times 40$, the population size *N* is approximately 4000. The curve obtained from the compartmental model and shown in Fig. 14 is shown with continuous blue line. (Color figure online)

Now we investigate the effect of the parameters $\tau _\mathrm{cwp}$, representing the relative infection rate within workplaces, and the size of workplaces. The left panel in Fig. 16 shows the $(R(\infty ), I_{max })$ pairs as $\tau _\mathrm{cwp}$ is varied from zero, corresponding to closed workplaces, to $\tau _\mathrm{_h}$, the relative infection rate at homes. We can observe that the averages (the black diamonds) form a nearly horizontal curve, i.e. $I_{max }$ is slightly affected by this infection rate. The maximum value of *I*, belonging to a given final size, is roughly three or four times larger for the network propagation than for the propagation in the compartmental model.

The right panel in Fig. 16 shows these pairs as the workplace size is varied from 4 to 18. The curve formed by the averages (black diamonds) is similar to that given by the compartmental model. We can observe that for larger work places the averages get closer to the curve given by the compartmental model.

Since we have seen in Fig. 10 that closing a part of the workplaces does not have a strong effect on the propagation, we do not consider this parameter here.

#### The effect of community places and the geometric structure

**Fig. 17:**
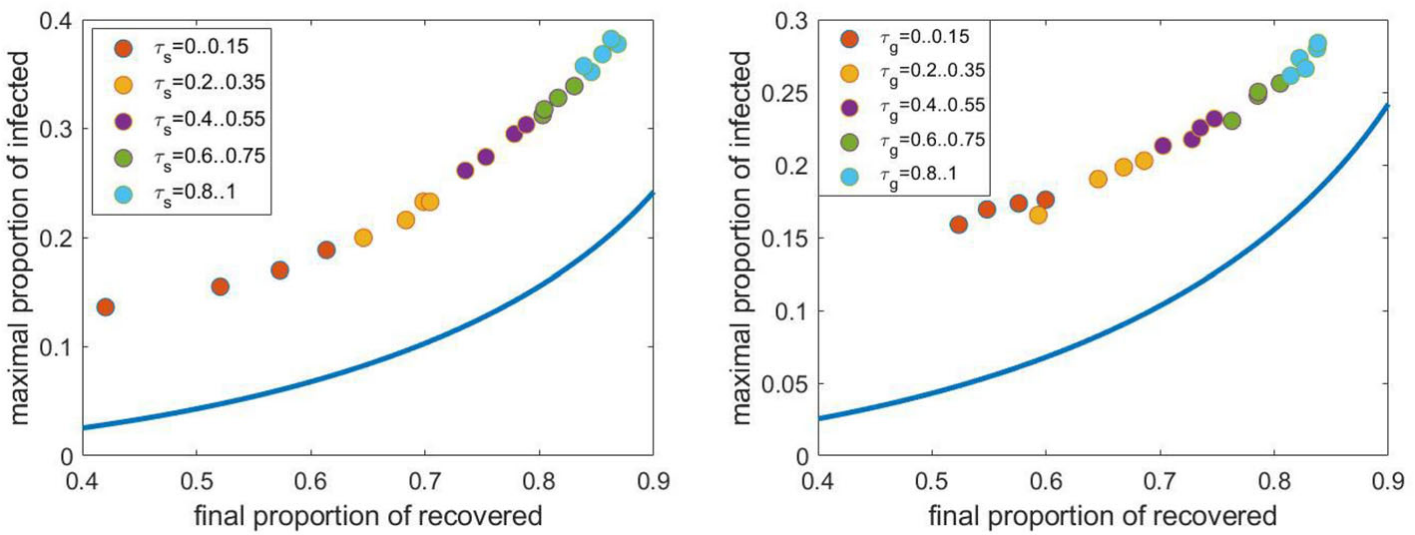
The pairs $(R(\infty ), \max I)$ obtained from stochastic simulation as $\tau _\mathrm{s}$ (left panel) and $\tau _\mathrm{g}$ (right panel) is varied. The average of the 10 pairs computed for a given network is shown. Other parameters are fixed according to Sect. 2.2, the household network is a grid of size $40\times 40$, the population size *N* is approximately 4000. The curve obtained from the compartmental model and shown in Fig. 14 is shown with continuous blue line. (Color figure online)

Now we investigate the effect of the parameters $\tau _\mathrm{s}$ and $\tau _\mathrm{g}$, representing the relative infection rates at public places and between households. Now only the average of the pairs $(R(\infty ), I_{max })$ is plotted. The left panel in Fig. 17 shows these pairs as $\tau _\mathrm{s}$ is varied from zero, corresponding to closed public places, to $\tau _\mathrm{_h}$, the relative infection rate at homes. We can observe that the points form a curve similar to that given by the compartmental model, but lying to the left of this. The maximum value of *I*, belonging to a given final size, is roughly two or three times larger for the network compared to compartmental model.

The right panel in Fig. 17 shows these pairs as $\tau _\mathrm{g}$ is varied from zero, corresponding to separated households, to $\tau _\mathrm{_h}$, the relative infection rate at homes. The curve formed by the points is similar to that given by the compartmental model. We can observe that for larger values of $\tau _\mathrm{g}$ the averages get closer to the curve given by the compartmental model.

#### The combined effect of all parameters

**Fig. 18:**
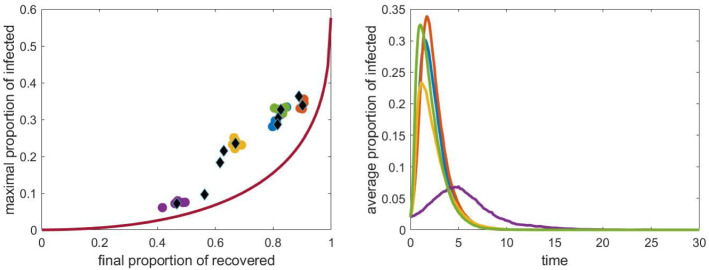
Left panel: the pairs $(R(\infty ), \max I)$ obtained from stochastic simulation for ten randomly chosen parameter sets. The range of the parameters is given in the text. The average of 5 simulations is shown with black diamonds. The household network is a grid of size $40\times 40$, the population size *N* is approximately 4000. The curve obtained from the compartmental model and shown in Fig. 14 is shown with continuous red line. Right panel: time dependence of *I* for each parameter set. (Color figure online)

Finally, we varied the values of the parameters together. (In the previous sections one parameter was varied, while the values of the others were fixed.) The relative infection rates across the different layers were chosen uniformly at randomly from the interval [0, 1], the sizes of the cliques in schools were chosen uniformly at randomly from the numbers 4, 6, 10, 15, 25, the school sizes were fixed at 200, the work place sizes were 4 and 18 and the capacity of the public places was one of the numbers 100, 150, 200, 250, 300. Ten networks were created randomly for each parameter set and five stochastic simulations were run on each graph until the propagation process finished. Then we determined the final epidemic size, $R(\infty )$ and the maximum value of the number of infected nodes, $I_{max }$. The results are presented in Fig. 18. The black diamonds in the left panel show the average of five simulations. For some cases, the results of each simulation are shown with coloured dots. The corresponding time dependence of *I* is shown in the right panel. We can observe that the ten networks and parameter sets yield very different $(R(\infty ), I_{max })$ pairs, however, they form a curve similar to that obtained from the compartmental model, but lying to the left of this.

## Discussion

In this paper we studied the impact of population contact structure, in the form of a multilayer network, on the time course and the outcome of an SIR epidemic. The contact network was comprised of multiple layers including households, schools and workplaces, spatial/physical embedding of the individuals and casual contacts during shopping, visits to general practice, communal play areas etc. While this is an idealised model it nevertheless incorporates some of the principal routes of spread for infectious disease such as COVID-19. In this study, we mainly used simulations since the networks are highly structured featuring clustering of contacts and spatial structure and thus are not amenable to for the development of good quality mean-field approximations.

Based on our interpretation of contact structure and assumptions behind our multilayer network we found that compared to the compartmental model and fixed final epidemic size, epidemics on networks lead to higher values of peak epidemic prevalence. Intuitively, this is due to the heterogeneity in contact where highly connected nodes are found early and produce many infections early on (i.e. super-spreading events [22]), leading to high values of $I_{max }$. However, such nodes are few and after these have been infected the epidemic is now unfolding over the less-well connected part of the network. These nodes are harder to reach and therefore the final epidemic size will be smaller compered to the compartmental model case. This can be seen in the left panels of any of the Figs. 15, 16, 17 and 18, where fixing a value of $I_{max }$ for the network model leads to a higher final epidemic size in the compartmental model with the same peak prevalence. It could be the subject of further study how the network properties determine the curve formed by the pairs $(R(\infty ), \max I)$ in the above Figures.

For schools, the biggest impact was attained where the clique sizes were small and with little interaction with other cliques. Furthermore, even a weak connection of students belonging to different cliques can lead to a strong epidemic wave. Hence keeping different school cliques isolated, or in a bubble, seems to be an effective control measure. We found that workplaces had a small impact on the epidemic. The most important characteristic turned out to be the size of the work places. Closing the workplaces partially seems to have a little effect.

Of course, further refinements of the model could include care homes and other vulnerable parts of the society. Moreover, the epidemic dynamics can be made more realistic by adding extra stages to depart from the non-exponential infectious times or to model explicitly those that are symptomatic/asymptomatic and those that need hospitalisation with eventual recovery or death. Furthermore, the network itself could be made dynamic or adaptive [14, 23] since during an epidemic the contact structure is dynamic, especially with different types of lockdowns put in place for different amounts of time.
